# Correction: Evaluating epidemic forecasts in an interval format

**DOI:** 10.1371/journal.pcbi.1010592

**Published:** 2022-10-05

**Authors:** Johannes Bracher, Evan L. Ray, Tilmann Gneiting, Nicholas G. Reich

In subsection 3.2 two example values of the weighted interval score (a score for forecast accuracy described in the paper) were incorrect. The respective sentence should read:

"The WIS (with K = 11 as in the previous section), on the other hand, favors G as its quantiles are generally closer to the observed value y (WIS(F, 190) = 105.3, WIS(G, 190) = 88.9)."

These values had erroneously been given as (WIS(F, 190) = 103.9, WIS(G, 190) = 87.8).

The following code availability information was missing from the published article: code to reproduce Fig 1–6 has been made available at https://github.com/reichlab/proper-scores-comparison. All data used in this paper have been taken from the public cdc-flusight-ensemble repository https://github.com/FluSightNetwork/cdc-flusight-ensemble.

The dark green line shown in the middle right and bottom right panels of Fig 2 and the right panel of Fig 3 did not display the correct values. The authors have provided corrected versions here.

**Fig 2:**
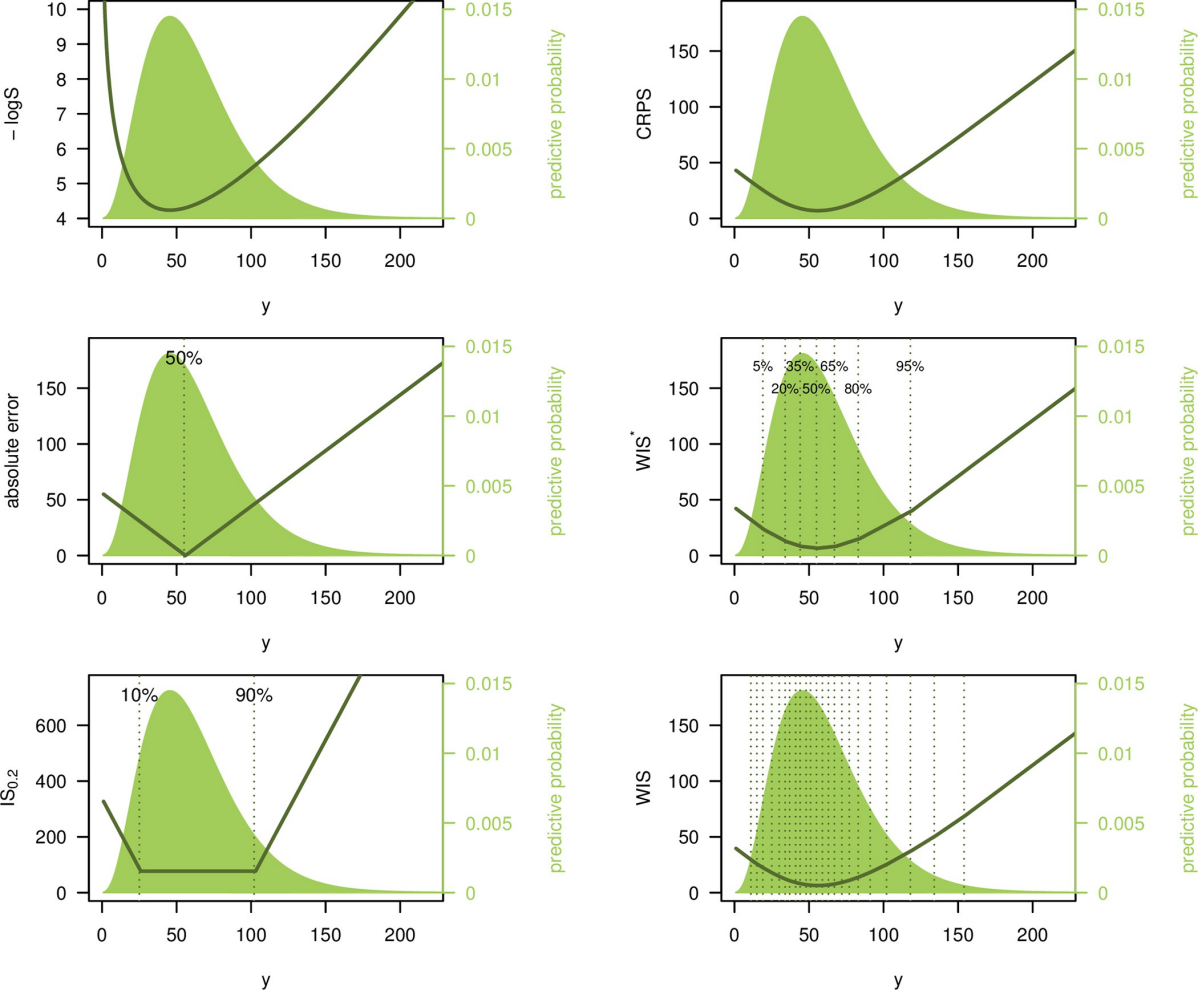
Illustration of different scoring rules. Logarithmic score, absolute error, interval score (with ***α* = 0.2**), CRPS, and 2 versions of the weighted interval score. These are denoted by WIS* (with ***K*** = **3**, ***α***_**1**_ **= 0.1, *α***_**2**_ **= 0.4, *α***_**3**_ **= 0.7**) and **WIS** (***K* = 11, *α***_**1**_ **= 0.02, *α***_**2**_ **= 0.05, *α***_**3**_ **= 0.1,…,*α***_**11**_ **= 0.9**). Scores are shown as a function of the observed value ***y***. The predictive distribution ***F*** is negative binomial with expectation 60 and size 4. Note that the top left panel shows the negative **logS**, i.e., −**logS**, which, like the other scores, is negatively oriented (smaller values are better).

**Fig 3:**
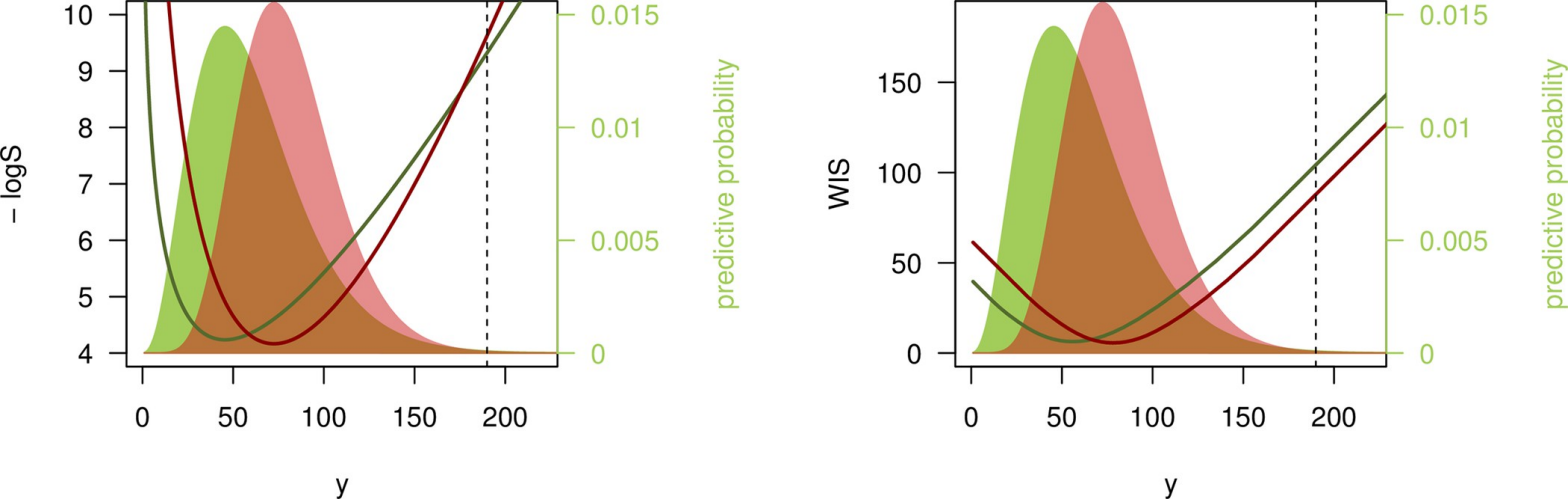
Disagreement between logarithmic score and WIS. Negative logarithmic score and weighted interval score (with ***α***_**1**_ **= 0.02, *α***_**2**_ **= 0.05, *α***_**3**_ **= 0.1,…,*α***_**11**_ **= 0.9**) as a function of the observed value ***y***. The predictive distributions ***F*** (green) and ***G*** (red) are negative binomials with expectations ***μ***_***F***_ **= 60**, ***μ***_***G***_ **= 80** and sizes ***ψ***_***F***_ **= 4, *ψ***_***G***_ **= 10**. The black dashed line shows ***y*** = **190** as discussed in the text.
