# Supplementary material for: Evaluating epidemic forecasts in an interval format
Source: PLoS Comput Biol. 2021 Feb 12;17(2):e1008618. doi: 10.1371/journal.pcbi.1008618 (PMC7880475; doi:10.1371/journal.pcbi.1008618)
Supplement: S1 Text — (PDF) [file pcbi.1008618.s001.pdf]

Supplement (S1 Text) for "Evaluating epidemic forecasts in an interval format"

Johannes Bracher[1,2], Evan L. Ray[3], Tilmann Gneiting[2,4] and Nicholas G. Reich[3]

[1]Karlsruhe Institute of Technology (KIT), Chair of Econometrics and Statistics
[2]Heidelberg Institute for Theoretical Studies
[3]University of Massachusetts, School of Public Health and Health Sciences, Department of Biostatistics and Epidemiology
[4]Karlsruhe Institute of Technology (KIT), Institute for Stochastics

January 13, 2021

1 Relationship between quantile score, interval score and CRPS

The standard piecewise linear quantile score [1,2] for the level τ is defined as

$$\text{QS}_\tau(F, y) = 2 \times \{\mathbf{1}(y \leq q_\tau) - \tau\} \times (q_\tau - y),$$

where q_τ is the τ quantile of the forecast F and y is the observed outcome. It can be shown by some re-ordering of terms that the interval score of a central $(1-\alpha)$ PI can be computed from the quantile scores at levels $\alpha/2$ and $1-\alpha/2$ as

$$\text{IS}_\alpha(F, Y) = \frac{\text{QS}_{\alpha/2}(F, y) + \text{QS}_{1-\alpha/2}(F, y)}{\alpha}. \tag{S.1}$$

Interestingly, this is the only available proper interval score that is invariant under translation [3, Theorem 4], so that for a prediction horizon of one time unit, evaluations in terms of incident counts yield the same results as evaluations in terms of cumulative counts.

Moreover it is known [4,5] that

$$\begin{aligned} \text{CRPS}(F, y) &= \int_0^1 \text{QS}_\tau(F, y)\, \mathrm{d}\tau, \\ &\approx \frac{1}{2K+1} \times \sum_{k=1}^{2K+1} \text{QS}_{\tau_k}(F, y), \\ &= \frac{1}{2K+1} \times \sum_{k=1}^{2K+1} 2 \times \{\mathbf{1}(y \leq q_{\tau_k}) - \tau_k\} \times (q_{\tau_k} - y), \end{aligned} \tag{S.2}$$

with a large number of (approximately) equally spaced levels τ_k stretching the unit interval such that $\tau_1 < \dots < \tau_{K+1} = 1/2 < \dots < \tau_{2K+1}$. Note that expression (S.2) is the same as the alternative expression (4) for the WIS from the main text, where $\tau_k = \alpha_k/2$ and $\tau_{2K+2-k} = 1 - \alpha_k/2$ for $k = 1, \dots, K$.

Indeed, starting from the original definition of the WIS in equation (1) with weights $w_0 = 1/2$ and $w_k = \alpha_k/2$ for $k = 1, \dots, K$ as in equation (2) from the main text, using equation (S.1),

and noting that $\tau_{K+1} = 1/2$ and $q_{\tau_{K+1}} = m$ is the median, we see that

$$\begin{aligned}\mathrm{WIS}_{\alpha_{0:K}}(F, y) &= \frac{1}{2K+1} \times \left(|y - m| \ + \sum_{k=1}^{K} \alpha_k \times \mathrm{IS}_{\alpha_k}(F, y) \right) \\ &= \frac{1}{2K+1} \left(\mathrm{QS}_{\tau_{K+1}}(F, y) + \sum_{k=1}^{K} \left\{ \mathrm{QS}_{\tau_k}(F, y) + \mathrm{QS}_{\tau_{2K+2-k}}(F, y) \right\} \right) \\ &= \frac{1}{2K+1} \times \sum_{k=1}^{2K+1} 2 \times \{\mathbf{1}(y \le q_{\tau_k}) - \tau_k\} \times (q_{\tau_k} - y).\end{aligned}$$

As noted in Section 2.2, the τ_k we use in practice are not equally spaced in the tails (due to the addition of the quantiles at levels 0.01, 0.025, 0.975, and 0.99 forming the 95% and 98% prediction intervals). Relative to the CRPS, we thus put slightly more weight on the tails.

References

1. Gneiting T, Raftery AE. Strictly proper scoring rules, prediction, and estimation. Journal of the American Statistical Association. 2007;102(477):359–378. doi:10.1198/016214506000001437.

2. Gneiting T. Making and evaluating point forecasts. Journal of the American Statistical Association. 2011;106(494):746–762. doi:10.1198/jasa.2011.r10138.

3. Brehmer JR, Gneiting T. Scoring interval forecasts: Equal-tailed, shortest, and modal interval. Bernoulli. 2021; forthcoming. Preprint available at https://arxiv.org/abs/2007.05709.

4. Laio F, Tamea S. Verification tools for probabilistic forecasts of continuous hydrological variables. Hydrology and Earth System Sciences Discussions. 2007;11(4):1267–1277. doi:10.5194/hess-11-1267-2007

5. Gneiting T, Ranjan R. Comparing density forecasts using threshold- and quantile-weighted scoring rules. Journal of Business and Economic Statistics. 2011;29(3):411–422. doi:10.1198/jbes.2010.08110
